# Nanobeams with Internal Discontinuities: A Local/Nonlocal Approach

**DOI:** 10.3390/nano11102651

**Published:** 2021-10-09

**Authors:** Daniela Scorza, Sabrina Vantadori, Raimondo Luciano

**Affiliations:** 1 Department of Engineering, University of Naples Parthenope, Centro Direzionale Isola C4, 80143 Naples, Italy; daniela.scorza@uniparthenope.it (D.S.); raimondo.luciano@uniparthenope.it (R.L.); 2 Department of Engineering and Architecture, University of Parma, Parco Area delle Scienze 181/A, 43124 Parma, Italy

**Keywords:** energy release rate, internal discontinuity, mixture parameter, nanobeam, stress-driven integral model, stress intensity factor

## Abstract

The aim of the present work is to extend the two-phase local/nonlocal stress-driven integral model (SDM) to the case of nanobeams with internal discontinuities: as a matter of fact, the original formulation avoids the presence of any discontinuities. Consequently, here, for the first time, the problem of an internal discontinuity is addressed by using a convex combination of both local and nonlocal phases of the model by introducing a mixture parameter. The novel formulation here proposed was validated by considering six case studies involving different uncracked nanobeams by varying the constrains and the loading configurations, and the effect of nonlocality on the displacement field is discussed. Moreover, a centrally-cracked nanobeam, subjected to concentrated forces at the crack half-length, was studied. The size-dependent Mode I fracture behaviour of the cracked nanobeam was analysed in terms of crack opening displacement, energy release rate, and stress intensity factor, showing the strong dependency of the above fracture properties on both dimensionless characteristic length and mixture parameter values.

## 1. Introduction

In the last few decades, structures at the nanoscale level have gained an increasing interest in engineering applications. The reason for such concern from the scientific community is mainly due to the outstanding mechanical, electrical, and thermal properties resulting from the nanoscale sizes [1].

The behaviour of materials at the nanoscale level is significantly different from that exhibited by the same materials at the macroscale level [2,3,4,5,6]. In order to both analyse the so-called size effect and properly evaluate the size-dependent properties, two approaches may be followed: experimental characterisation [7,8,9] and theoretical modelling [10,11,12,13,14,15,16,17,18,19,20,21,22,23,24,25,26]. For instance, the tensile yield strength of a gradient nano-grained (GNG) surface layer in a bulk coarse-grained (CG) rod of a face-centred cubic Cu was investigated by Fang et al. [7], who observed an increment of strength of about 100% with respect to that of a CG Cu. A study on ductility and strain hardening on a sandwich sheet structure, composed by a CG core between two GNG layers, was conducted by Wu et al. [8].Also, the fracture properties of single-crystalline copper nanowires have been investigated by performing uniaxial tensile tests through a micromechanical device inside a scanning electron microscope chamber [9]. It was observed that the fracture strength was much higher than that of bulk copper and that both ductile and brittle-like fracture modes were present in the same batch of fabricated nanowires, depending on their diameters [9].

However, despite their high level of reliability, performing experimental tests at the nanoscale may be quite expensive and time consuming, leading to often prefer theoretical models that are reliable and low-cost tool to estimate the behaviour of the nanomaterials. As a matter of fact, several theoretical models have been proposed aiming to capture the small-scale effect on the static [10,11,12,13,14,15] and dynamic responses [11,12,13,18,19,20,21,22,23,24,25], instability [12,24,25,26,27,28] and fracture behaviour [10,29,30] of nanomaterials at both the micro- or nano-scale level. In addition, the stress-driven nonlocal integral model (SDM) is available in the literature [31,32,33,34,35,36,37,38].

Such a nonlocal theory of elasticity is based on the well-known Eringen strain-driven model [39,40], but, in order to overcome the solution inconsistencies of the original model due to the incompatibility between nonlocal constitutive law and equilibrium requirements [41,42], the role of the strain field has been swapped with that of stress field. As a matter of fact, the SDM assumes that the strains at a point of a body are the function of the stresses at all points of the body. Consequently, the elastic strain field is defined by an integral convolution between the elastic stress field and a suitable averaging kernel as well as the associated constitutive boundary conditions, which are expressed in terms of strains [31,32], leading to a well-posed elastostatic problem. The model has been successfully applied to study nanobeams subjected to different loading configurations such as bending [31,32,34], axial load [43], torsion [44], buckling [35,36,37,45], and free vibrations [33,38,46].

A further development of the SDM has led to the implementation of a mixture parameter aiming to improve the applicability and accuracy of the model, named two-phase local/nonlocal stress-driven model [47,48], defined by a convex combination of both local and nonlocal phases. The two-phase local/nonlocal SDM has been applied to study free bars under uniform tension, nanobeams under bending loading [47,48], and nanobeams under free vibrations [49].

All the works above cited refer to continuous nanobeams subjected to smooth loading distributions or loads applied at the beam ends, since the above two-phase local/nonlocal model does not allow to consider the presence of discontinuities, which are very common in engineering applications. Such discontinuities may be caused, for instance, by concentrated forces and/or couples, non-uniform distributed loads, discontinuity in the beam geometry, internal supports/constrains, and so on.

The novelty of the present paper is to extend the two-phase local/nonlocal stress-driven integral model formulation in the case of problems with internal discontinuities. Here, for the first time, such a problem of internal discontinuities is addressed by using a convex combination of both local and nonlocal phases of the model (which the nonlocal phase is modelled according to the SDM) by introducing the mixture parameter *α*.

In particular, in Section 2, the novel procedure is presented [50]. The above formulation, applied to different static schemes and loading configurations of uncracked nanobeams, is presented in Section 3, where the displacement field is analysed and the effect of both mixture and nonlocal parameters are discussed. Moreover, a centrally-cracked nanobeam (CCN), subjected to concentrated forces at the crack half-length, is studied in Section 4, where the novel procedure is employed to numerically investigate the fracture behaviour of the CCN and the effect of both mixture and nonlocal parameters on the energy release rate and the stress intensity factor. Finally, the main conclusions are reported in Section 5.

## 2. The Two-Phase Local/Nonlocal SDM in the Presence of Internal Discontinuities

### 2.1. Integral Formulation

**Figure 1:**
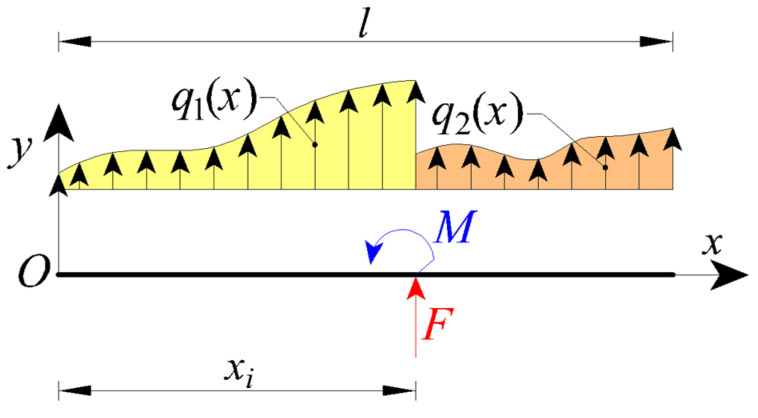
Nanobeam containing some loading discontinuities.

Let us consider the nanobeam shown in Figure 1 with a length equal to *l* and the reference frame xy.

The nanobeam is subjected to a loading configuration presenting internal discontinuities (due, for example, to concentrated forces and/or couples or due to discontinuity of non-uniform distributed loads) at the material point characterised by $x=x_{i}$.

The nanobeam is subdivided into two parts (the left one, also named as the first part, with $0\leq x\leq x_{i}$ and the right one, also named as the second part, with $x_{i}\leq x\leq l$), so that each nanobeam part does not present any internal discontinuity and its transversal displacement could be described by a continuous function.

According to the two-phase local/nonlocal SDM [48], the flexural curvature χ(x) of a nanobeam without discontinuity is defined as the integral convolution of the bending moment, M(x), by introducing a suitable kernel function as reported in the following:

(1) $$\chi \left(x\right)=\alpha \frac{M\left(x\right)}{IE}+\left(1-\alpha \right)\int_{0}^{l}\psi \left(x-t,L_{c}\right)\frac{M\left(t\right)}{IE}dt$$

where IE is the nanobeam bending stiffness, and $\psi \left(x-t,L_{c}\right)$ is an averaging kernel depending on the small scale parameter $L_{c}$, named characteristic or internal material length. α is the mixture parameter ranging from 0.0 to 1.0, so that for α=0.0, the full nonlocal stress-driven model is recovered, whereas for α=1.0, the classical Bernoulli–Euler model is obtained.

Let us consider an internal discontinuity in correspondence of $x_{i}$ (see Figure 1). In such a case, χ(x) is given by:

(2) $$\chi \left(x\right)=\alpha \frac{\left[M_{1}\left(x\right)+M_{2}\left(x\right)\right]}{IE}+\left(1-\alpha \right)\left[\int_{0}^{x_{i}}\psi \left(x-t,L_{c}\right)\frac{M_{1}\left(t\right)}{IE}dt+\int_{x_{i}}^{l}\psi \left(x-t,L_{c}\right)\frac{M_{2}\left(t\right)}{IE}dt\right]$$

where $M_{1}\left(x\right)$ is the bending moment acting on the first part, and $M_{2}\left(x\right)$ acts on the second part of the nanobeam.

### 2.2. Differential Formulation

In order to compute the transversal displacement field, it is more convenient to not directly solve Equation (2), but to employ its equivalent differential formulation, by following the procedure proposed by Caporale et al. in ref. [50] for the original full nonlocal SDM and here developed, instead, for the two-phase local/nonlocal SDM.

The curvature along the nanobeam is given by:

(3a) $$\chi_{1}\left(x\right)=\alpha \frac{M_{1}\left(x\right)}{IE}+\left(1-\alpha \right)\left[\chi_{1,l}\left(x\right)+\chi_{1,r}\left(x\right)+\chi_{1,2}\left(x\right)\right]\;\mathrm{with}\;0\leq x\leq x_{i}$$

(3b) $$\chi_{2}\left(x\right)=\alpha \frac{M_{2}\left(x\right)}{IE}+\left(1-\alpha \right)\left[\chi_{2,l}\left(x\right)+\chi_{2,r}\left(x\right)+\chi_{2,1}\left(x\right)\right]\;\mathrm{with}\;x_{i}\leq x\leq l$$

with:

(4a) $$\chi_{1,l}\left(x\right)=\int_{0}^{x}\exp\left[\frac{\left(t-x\right)}{L_{c}}\right]\frac{1}{2L_{c}}\frac{M_{1}\left(t\right)}{IE}dt$$

(4b) $$\chi_{1,r}\left(x\right)=\int_{x}^{x_{i}}\exp\left[\frac{\left(x-t\right)}{L_{c}}\right]\frac{1}{2L_{c}}\frac{M_{1}\left(t\right)}{IE}dt$$

(4c) $$\chi_{1,2}\left(x\right)=\int_{x_{i}}^{l}\exp\left[\frac{\left(x-t\right)}{L_{c}}\right]\frac{1}{2L_{c}}\frac{M_{2}\left(t\right)}{IE}dt$$

and

(5a) $$\chi_{2,l}\left(x\right)=\int_{x_{i}}^{x}\exp\left[\frac{\left(t-x\right)}{L_{c}}\right]\frac{1}{2L_{c}}\frac{M_{2}\left(t\right)}{IE}dt$$

(5b) $$\chi_{2,r}\left(x\right)=\int_{x}^{l}\exp\left[\frac{\left(x-t\right)}{L_{c}}\right]\frac{1}{2L_{c}}\frac{M_{2}\left(t\right)}{IE}dt$$

(5c) $$\chi_{2,1}\left(x\right)=\int_{0}^{x_{i}}\exp\left[\frac{\left(t-x\right)}{L_{c}}\right]\frac{1}{2L_{c}}\frac{M_{1}\left(t\right)}{IE}dt$$

Let us consider only the first part of the nanobeam, since for the second part, analogous equations can be obtained.

First, the first, $\chi_{1}^{\left(1\right)}$, and second, $\chi_{1}^{\left(2\right)}$, derivatives of Equation (3a) are developed, that is:

(6) $$\begin{array}{ll} \chi_{1}^{\left(1\right)}\left(x\right) & =\alpha \frac{M_{1}^{\left(1\right)}\left(x\right)}{IE}+\left(1-\alpha \right)\left[\chi_{1,l}^{\left(1\right)}\left(x\right)+\chi_{1,r}^{\left(1\right)}\left(x\right)+\chi_{1,2}^{\left(1\right)}\left(x\right)\right]=\\ & =\alpha \frac{M_{1}^{\left(1\right)}\left(x\right)}{IE}+\left(1-\alpha \right)\left[-\frac{1}{L_{c}}\chi_{1,l}\left(x\right)+\frac{1}{L_{c}}\chi_{1,r}\left(x\right)+\frac{1}{L_{c}}\chi_{1,2}\left(x\right)\right] \end{array}$$

being

(7a) $$\chi_{1,l}^{\left(1\right)}\left(x\right)=\frac{d\chi_{1,l}\left(x\right)}{dx}=\frac{1}{L_{c}}\left[\frac{M_{1}\left(x\right)}{2IE}-\chi_{1,l}\left(x\right)\right]$$

(7b) $$\chi_{1,r}^{\left(1\right)}\left(x\right)=\frac{d\chi_{1,r}\left(x\right)}{dx}=\frac{1}{L_{c}}\left[\chi_{1,l}\left(x\right)-\frac{M_{1}\left(x\right)}{2IE}\right]$$

(7c) $$\chi_{1,2}^{\left(1\right)}\left(x\right)=\frac{d\chi_{1,r}\left(x\right)}{dx}=\frac{1}{L_{c}}\left[\chi_{1,2}\left(x\right)\right]$$

and

(8) $$\begin{array}{ll} \chi_{1}^{\left(2\right)}\left(x\right) & =\alpha \frac{M_{1}^{\left(2\right)}\left(x\right)}{IE}+\left(1-\alpha \right)\frac{1}{L_{c}^{2}}\left[-\frac{M_{1}\left(x\right)}{IE}+\chi_{1,l}\left(x\right)+\chi_{1,r}\left(x\right)+\chi_{1,2}\left(x\right)\right]=\\ & =\alpha \frac{M_{1}^{\left(2\right)}\left(x\right)}{IE}+\frac{1}{L_{c}^{2}}\left\{-\frac{M_{1}\left(x\right)}{IE}+\underset{\chi_{1}\left(x\right)}{\underbrace{\alpha \frac{M_{1}\left(x\right)}{IE}+\left(1-\alpha \right)\left[\chi_{1,l}\left(x\right)+\chi_{1,r}\left(x\right)+\chi_{1,2}\left(x\right)\right]}}\right\} \end{array}$$

Then, according to the model, the differential equation for the first part of the beam is obtained by imposing Equation (8) equal to 0:

(9) $$\chi_{1}^{\left(2\right)}\left(x\right)-\frac{1}{L_{c}^{2}}\chi_{1}\left(x\right)=\alpha \frac{M_{1}^{\left(2\right)}\left(x\right)}{IE}-\frac{1}{L_{c}^{2}}\frac{M_{1}\left(x\right)}{IE}$$

and similarly, the differential equation for the second part of the beam is given by:

(10) $$\chi_{2}^{\left(2\right)}\left(x\right)-\frac{1}{L_{c}^{2}}\chi_{2}\left(x\right)=\alpha \frac{M_{2}^{\left(2\right)}\left(x\right)}{IE}-\frac{1}{L_{c}^{2}}\frac{M_{2}\left(x\right)}{IE}$$

In order to compute a consistent solution of the above problem, constitutive boundary conditions and constitutive continuity conditions have to be derived. Let us start by considering Equation (3a) evaluated in x=0 and $x=x_{i}$:

(11a) $$\chi_{1}\left(0\right)=\alpha \frac{M_{1}\left(0\right)}{IE}+\left(1-\alpha \right)\left[\chi_{1,r}\left(0\right)+\chi_{1,2}\left(0\right)\right]$$

(11b) $$\chi_{1}\left(x_{i}\right)=\alpha \frac{M_{1}\left(x_{i}\right)}{IE}+\left(1-\alpha \right)\left[\chi_{1,l}\left(x_{i}\right)+\chi_{1,2}\left(x_{i}\right)\right]$$

being: $\chi_{1,l}\left(0\right)=\chi_{1,r}\left(x_{i}\right)=0$. Then, let us consider Equation (6) in x=0 and $x=x_{i}$, and by exploiting Equation (11a,b), $\chi_{1}^{\left(1\right)}$ is given by:

(12a) $$\chi_{1}^{\left(1\right)}\left(0\right)=\alpha \frac{M_{1}^{\left(1\right)}\left(0\right)}{IE}+\frac{\left(1-\alpha \right)}{L_{c}}\left[\chi_{1,r}\left(0\right)+\chi_{1,2}\left(0\right)\right]=\alpha \frac{M_{1}^{\left(1\right)}\left(0\right)}{IE}+\frac{1}{L_{c}}\chi_{1}\left(0\right)-\frac{\alpha }{L_{c}}\frac{M_{1}\left(0\right)}{IE}$$

(12b) $$\begin{array}{ll} \chi_{1}^{\left(1\right)}\left(x_{i}\right) & =\alpha \frac{M_{1}^{\left(1\right)}\left(x_{i}\right)}{IE}+\frac{\left(1-\alpha \right)}{L_{c}}\left[-\chi_{1,l}\left(x_{i}\right)+\chi_{1,2}\left(x_{i}\right)\right]=\\ & =\alpha \frac{M_{1}^{\left(1\right)}\left(x_{i}\right)}{IE}-\frac{1}{L_{c}}\chi_{1}\left(x_{i}\right)+\frac{\alpha }{L_{c}}\frac{M_{1}\left(x_{i}\right)}{IE}+\frac{2\left(1-\alpha \right)}{L_{c}}\chi_{1,2}\left(x_{i}\right) \end{array}$$

By separating the variables, Equation (12a,b) can be rewritten as:

(13a) $$\chi_{1}^{\left(1\right)}\left(0\right)-\frac{1}{L_{c}}\chi_{1}\left(0\right)=\alpha \frac{M_{1}^{\left(1\right)}\left(0\right)}{IE}-\frac{\alpha }{L_{c}}\frac{M_{1}\left(0\right)}{IE}$$

(13b) $$\chi_{1}^{\left(1\right)}\left(x_{i}\right)+\frac{1}{L_{c}}\chi_{1}\left(x_{i}\right)=\alpha \frac{M_{1}^{\left(1\right)}\left(x_{i}\right)}{IE}+\frac{\alpha }{L_{c}}\frac{M_{1}\left(x_{i}\right)}{IE}+\frac{2\left(1-\alpha \right)}{L_{c}}\chi_{1,2}\left(x_{i}\right)$$

representing Equation (13a) the constitutive boundary condition, and Equation (13b) the constitutive continuity condition for the first part of the beam. Similarly, the constitutive boundary and continuity conditions for the second part of the beam are:

(14a) $$\chi_{2}^{\left(1\right)}\left(l\right)+\frac{1}{L_{c}}\chi_{2}\left(l\right)=\alpha \frac{M_{2}^{\left(1\right)}\left(l\right)}{IE}+\frac{\alpha }{L_{c}}\frac{M_{2}\left(l\right)}{IE}$$

(14b) $$\chi_{2}^{\left(1\right)}\left(x_{i}\right)-\frac{1}{L_{c}}\chi_{2}\left(x_{i}\right)=\alpha \frac{M_{2}^{\left(1\right)}\left(x_{i}\right)}{IE}-\frac{\alpha }{L_{c}}\frac{M_{2}\left(x_{i}\right)}{IE}-\frac{2\left(1-\alpha \right)}{L_{c}}\chi_{2,1}\left(x_{i}\right)$$

### 2.3. Solution

Usually, it is more convenient to write the governing equations not in terms of curvature (see Equation (3a,b)), but in terms of transversal displacement, v(x), by both exploiting the well-known relationship $\chi \left(x\right)=v_{}^{\left(2\right)}\left(x\right)$, and deriving Equation (3a,b) two times with respect to the variable x:

(15a) $$v_{1}^{\left(6\right)}\left(x\right)-\frac{1}{L_{c}^{2}}v_{1}^{\left(4\right)}\left(x\right)=\alpha \frac{M_{1}^{\left(4\right)}\left(x\right)}{IE}-\frac{1}{L_{c}^{2}}\frac{M_{1}^{\left(2\right)}\left(x\right)}{IE}$$

(15b) $$v_{2}^{\left(6\right)}\left(x\right)-\frac{1}{L_{c}^{2}}v_{2}^{\left(4\right)}\left(x\right)=\alpha \frac{M_{2}^{\left(4\right)}\left(x\right)}{IE}-\frac{1}{L_{c}^{2}}\frac{M_{2}^{\left(2\right)}\left(x\right)}{IE}\;$$

where $v_{1}$ is the displacement of the first part of the nanobeam, and $v_{2}$ is that of the second part.

The two constitutive boundary conditions (CBCs) in x=0 and x=l are given by:

(16a) $$v_{1}^{\left(3\right)}\left(0\right)-\frac{1}{L_{c}}v_{1}^{\left(2\right)}\left(0\right)=\alpha \frac{M_{1}^{\left(1\right)}\left(0\right)}{IE}-\frac{\alpha }{L_{c}}\frac{M_{1}\left(0\right)}{IE}$$

(16b) $$v_{2}^{\left(3\right)}\left(l\right)+\frac{1}{L_{c}}v_{2}^{\left(2\right)}\left(l\right)=\alpha \frac{M_{2}^{\left(1\right)}\left(l\right)}{IE}+\frac{\alpha }{L_{c}}\frac{M_{2}\left(l\right)}{IE}$$

whereas the two constitutive continuity conditions (CCCs) in $x=x_{i}$ are:

(17a) $$v_{1}^{\left(3\right)}\left(x_{i}\right)+\frac{1}{L_{c}}v_{1}^{\left(2\right)}\left(x_{i}\right)=\alpha \frac{M_{2}^{\left(1\right)}\left(x_{i}\right)}{IE}+\frac{\alpha }{L_{c}}\frac{M_{1}\left(x_{i}\right)}{IE}+\frac{2\left(1-\alpha \right)}{L_{c}}\chi_{1,2}\left(x_{i}\right)$$

(17b) $$v_{2}^{\left(3\right)}\left(x_{i}\right)-\frac{1}{L_{c}}v_{2}^{\left(2\right)}\left(x_{i}\right)=\alpha \frac{M_{2}^{\left(1\right)}\left(x_{i}\right)}{IE}-\frac{\alpha }{L_{c}}\frac{M_{2}\left(x_{i}\right)}{IE}-\frac{2\left(1-\alpha \right)}{L_{c}}\chi_{2,1}\left(x_{i}\right)$$

By solving the sixth-order differential equations (see Equation (15a,b)), the general integral solution depends on twelve integration constants. Therefore, the solving system is obtained by imposing twelve boundary conditions: the two CBCs given by Equation (16a,b), the two CCCs given by Equation (17a,b), and eight suitable kinematic/static boundary conditions at the nanobeam ends (x=0 and x=l) and at the internal point $x=x_{i}$.

In the following, six examples of nanobeams, characterised by an internal loading discontinuity, are solved.

## 3. Applications: Uncracked Nanobeams

### 3.1. Nanobeams with a Concentrated Force in the Midsection

**Figure 2:**
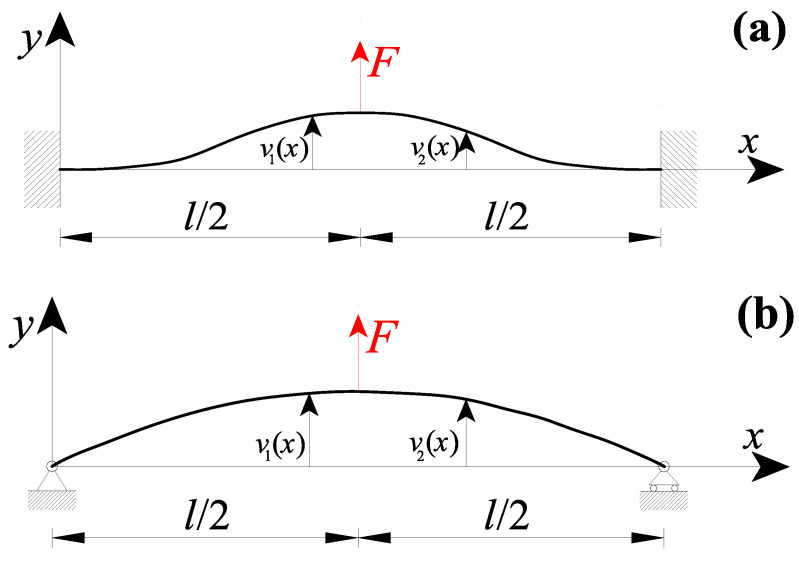
Nanobeam containing a loading discontinuity in the midsection due to a concentrated force F, with the beam ends: (**a**) double-clamped and (**b**) simply-supported.

Figure 2a shows a nanobeam of a length *l* with clamped extremities and subjected to a concentrated force F in the midsection (i.e., x=l/2). According to the equations presented in the above section, the nanobeam transversal displacement may be obtained by considering, in addition to both Equation (16a,b) (CBCs) and Equation (17a,b) (CCCs), the following conditions:

(i) six kinematic boundary conditions (KBCs):
  (18a) $$v_{1}^{}(0)=0$$
  (18b) $$v_{1}^{\left(1\right)}(0)=0$$
  (18c) $$v_{2}(l)=0$$
  (18d) $$v_{2}^{\left(1\right)}(l)=0$$
  (18e) $$v_{1}\left(l/2\right)=v_{2}\left(l/2\right)$$
  (18f) $$v_{1}^{\left(1\right)}\left(l/2\right)=v_{2}^{\left(1\right)}\left(l/2\right)$$
(ii) two static boundary conditions (SBCs):
  (19a) $$v_{1}^{\left(3\right)}\left(l/2\right)-L_{c}^{2}v_{1}^{\left(5\right)}\left(l/2\right)+\frac{F}{IE}=v_{2}^{\left(3\right)}\left(l/2\right)-L_{c}^{2}v_{2}^{\left(5\right)}\left(l/2\right)$$
  (19b) $$v_{1}^{\left(2\right)}\left(l/2\right)-L_{c}^{2}v_{1}^{\left(4\right)}\left(l/2\right)=v_{2}^{\left(2\right)}\left(l/2\right)-L_{c}^{2}v_{2}^{\left(4\right)}\left(l/2\right)$$

The maximum deflection $v_{\max}$ is attained at the nanobeam half length (that is, for x=l/2). The nanobeam transversal displacement is more conveniently normalised with respect to the maximum deflection obtained according to the classical local Bernoulli–Euler solution, that is, $v_{1}^{*}(x)=v_{1}(x)/\left(Fl^{3}/192EI\right)$ and $v_{2}^{*}(x)=v_{2}(x)/\left(Fl^{3}/192EI\right)$.

**Figure 3:**
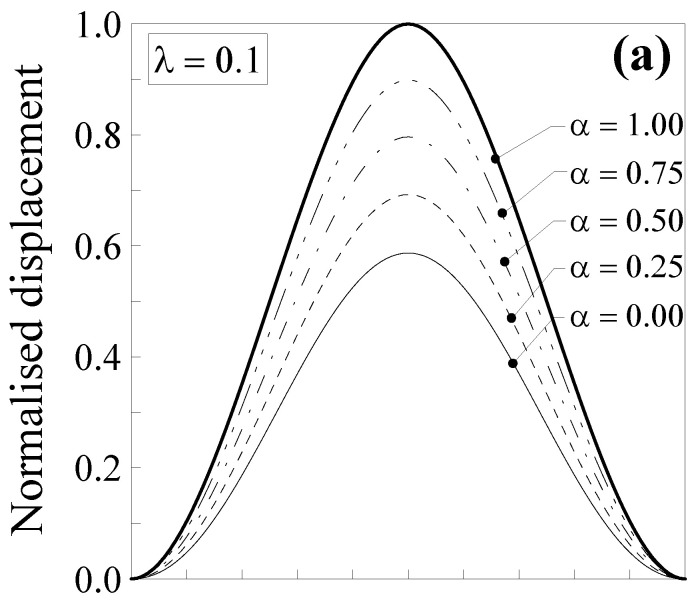

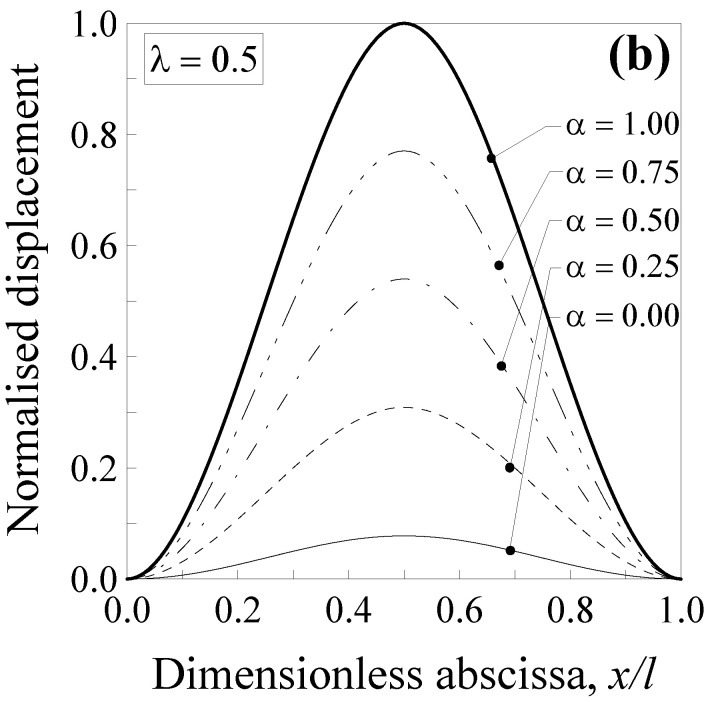
Double-clamped nanobeam (see Figure 2a): normalised transversal displacement against the dimensionless abscissa, x/l, for (**a**) λ=0.1 and (**b**) λ=0.5. Five different values of α were analysed.

In Figure 3, the normalised displacement is plotted against the dimensionless abscissa x/l. Five values of the mixture parameter were considered (that is, α=0.00, 0.25, 0.50, 0.75 and 1.00), together with two values of the dimensionless characteristic length, that is, $\lambda =L_{c}/l=0.1$ (Figure 3a) and λ=0.5 (Figure 3b).

It is interesting to observe that the normalised transversal displacement strongly depends on both the mixture parameter, α, and the dimensionless characteristic length, λ. The case with α=1.0 (thick line) corresponds to the case of the classical Bernoulli–Euler local problem. For a smaller value of α, the normalised displacement values are lower, showing a decrease of about 41% for λ=0.1 (Figure 3a) and of about 92% for λ=0.5 (Figure 3b) when α=0.0 (full nonlocal model) and x/l=0.5. Consequently, the nanobeam shows a stiffer behaviour with respect to a large-scale beam. Moreover, it is interesting to observe that by varying the mixture parameter between 0.0 and 1.0, the curves describing the normalised displacement stay between the thick and the thin ones. This allows us to improve the model applicability since α may be calibrated, in order to properly describe the behaviour of real nanostructures.

Analogously, Figure 2b shows a nanobeam of a length l, with simply-supported extremities, and subjected to a concentrated force F in the midsection. In such a case, the eight suitable boundary conditions are:

(i) four kinematic boundary conditions (KBCs):
  (20a) $$v_{1}\left(0\right)=0$$
  (20b) $$v_{2}\left(l\right)=0$$
  (20c) $$v_{1}\left(l/2\right)=v_{2}\left(l/2\right)$$
  (20d) $$v_{1}^{\left(1\right)}\left(l/2\right)=v_{2}^{\left(1\right)}\left(l/2\right)$$
(ii) two static boundary conditions (SBCs) at the nanobeam extremities:
  (21a) $$v_{1}^{\left(2\right)}\left(0\right)-L_{c}^{2}v_{1}^{\left(4\right)}\left(0\right)=0$$
  (21b) $$v_{2}^{\left(2\right)}\left(l\right)-L_{c}^{2}v_{2}^{\left(4\right)}\left(l\right)=0$$
(iii) and the two static boundary conditions (SBCs)at the internal discontinuity point of Equation (19a,b).

The maximum deflection $v_{\max}$ is attained at the nanobeam half length.

**Figure 4:**
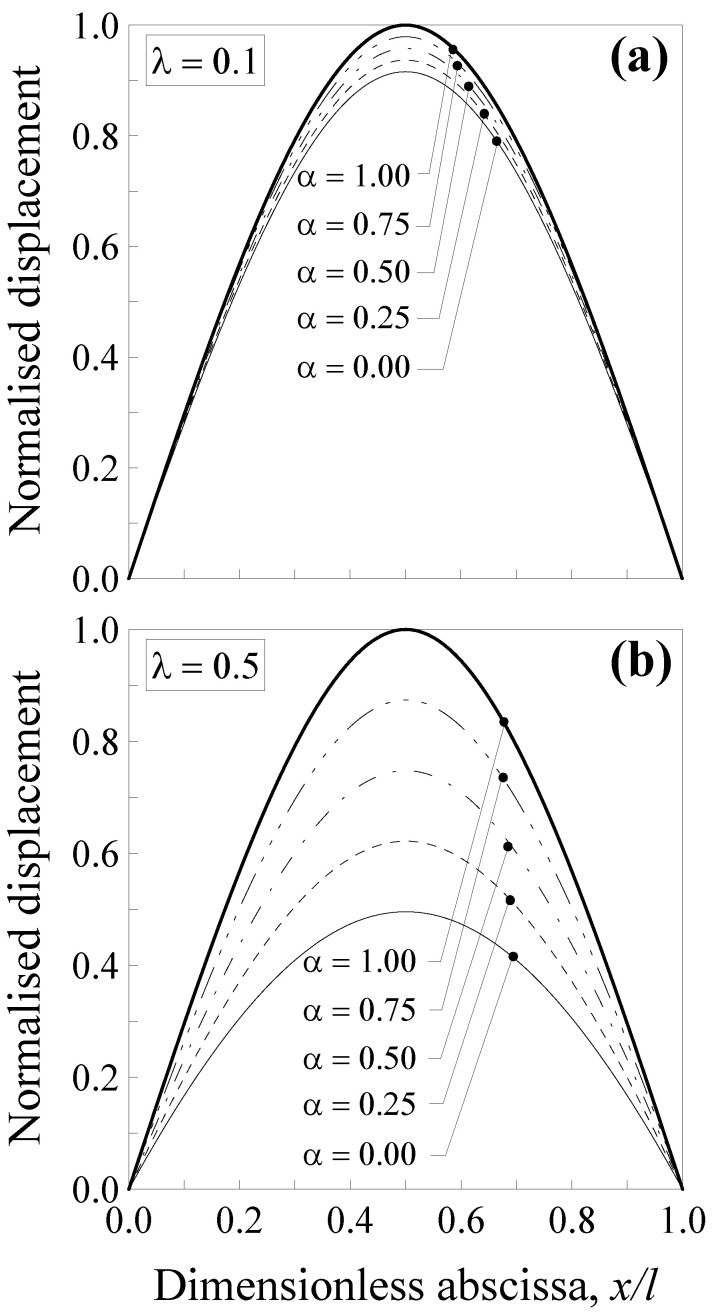
Simply-supported nanobeam (see Figure 2b): normalised transversal displacement against the dimensionless abscissa, x/l, for (**a**) λ=0.1 and (**b**) λ=0.5. Five different values of α were analysed.

Additionally, in this case, the transversal displacement is normalised with respect to the maximum deflection according to the classical local Bernoulli–Euler solution, that is, $v_{1}^{*}(x)=v_{1}(x)/\left(Fl^{3}/48EI\right)$ and $v_{2}^{*}(x)=v_{2}(x)/\left(Fl^{3}/48EI\right)$. Such a normalised displacement is plotted in Figure 4 against the dimensionless abscissa x/l for five values of the mixture parameter (that is, *α* = 0.00, 0.25, 0.5, 0.75 and 1.00) and two values of the dimensionless characteristic length, that is, λ=0.1 (Figure 4a) and λ=0.5 (Figure 4b).

It is noteworthy that the normalised displacement was less affected by nonlocality with respect to that of the double-clamped nanobeam (see Figure 3), since the decrease was equal to about 8% for λ=0.1 (Figure 4a) and of about 50% for λ=0.5 (Figure 4b) when α=0.0 and x/l=0.5. Moreover, it is interesting to observe that by varying the mixture parameter between 0.0 and 1.0, the curves describing the normalised displacement stay between the thick and the thin ones. This allows us to improve the model applicability since α may be calibrated in order to properly describe the behaviour of real nanostructures.

Additionally, in this case, the nanobeam shows a stiffer behaviour with respect to a large-scale beam.

### 3.2. Nanobeams with a Concentrated Couple in the Midsection

**Figure 5:**
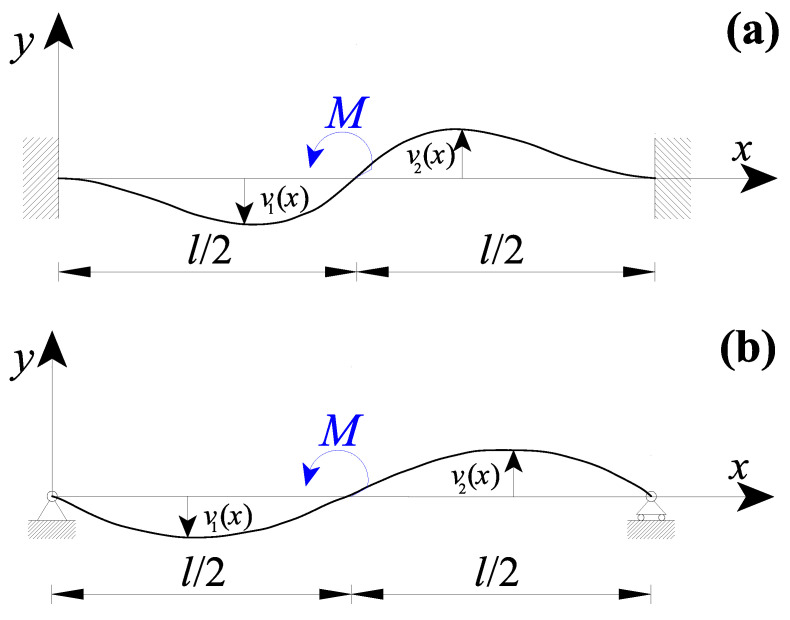
Nanobeam containing a loading discontinuity in the midsection due to a concentrated couple M, with the beam ends: (**a**) double-clamped and (**b**) simply-supported.

In the following case study, a double-clamped nanobeam with a length l and subjected to a concentrated couple M in the midsection (i.e., $x_{i}=l/2$) was considered (Figure 5a). The nanobeam transversal displacement may be obtained by considering in addition to both Equation (16a,b) (CBCs) and Equation (17a,b) (CCCs), the following conditions:

(i) the six kinematic boundary conditions (KBCs) of Equation (18a–f);
(ii) and two static boundary conditions (SBCs):
  (22a) $$v_{1}^{\left(3\right)}\left(l/2\right)-L_{c}^{2}v_{1}^{\left(5\right)}\left(l/2\right)=v_{2}^{\left(3\right)}\left(l/2\right)-L_{c}^{2}v_{2}^{\left(5\right)}\left(l/2\right)$$
  (22b) $$v_{1}^{\left(2\right)}\left(l/2\right)-L_{c}^{2}v_{1}^{\left(4\right)}\left(l/2\right)+\frac{M}{IE}=v_{2}^{\left(2\right)}\left(l/2\right)-L_{c}^{2}v_{2}^{\left(4\right)}\left(l/2\right)$$

The maximum deflection, as the absolute value, $\left|v_{\max}\right|$ is attained at the nanobeam points $x_{1}=l/3$ and $x_{2}=2l/3$.

The transversal displacement is normalised with respect to the absolute value of the maximum deflection according to the classical local Bernoulli–Euler solution, that is, $v_{1}^{*}(x)=v_{1}(x)/\left|M\cdot l^{2}/216EI\right|$ and $v_{2}^{*}(x)=v_{2}(x)/\left|M\cdot l^{2}/216EI\right|$.

**Figure 6:**
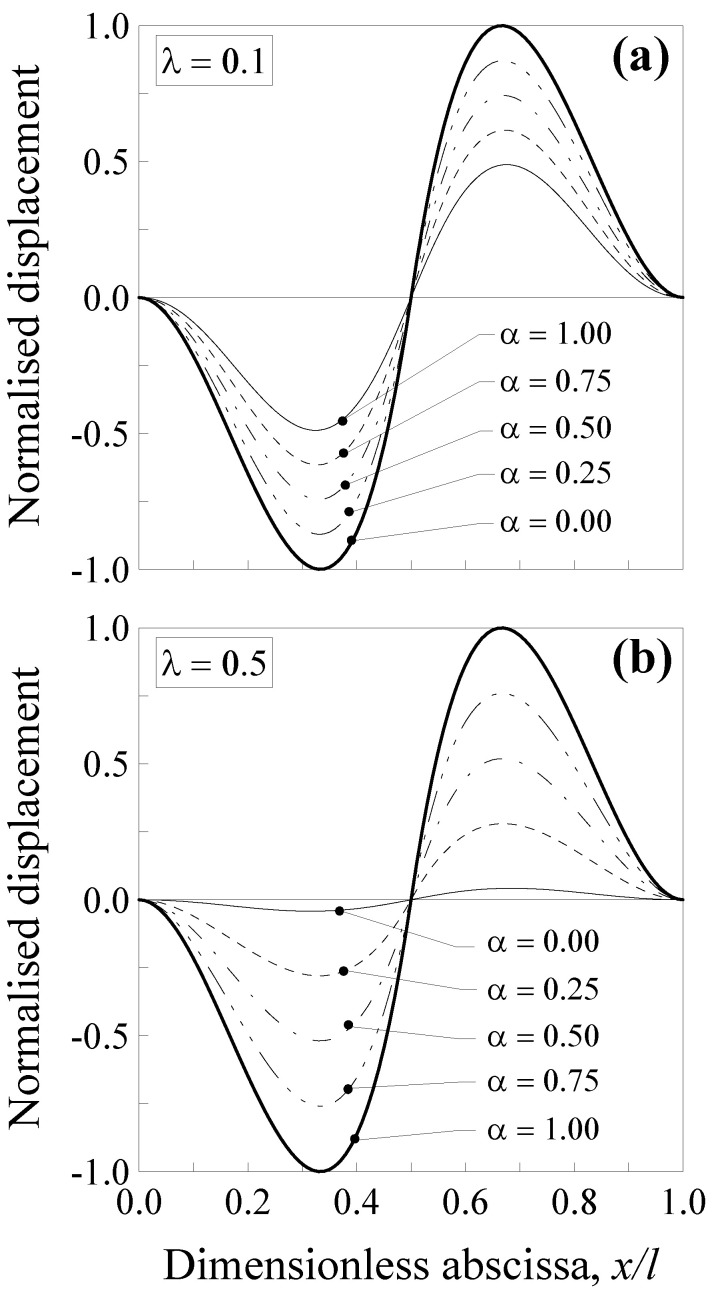
Double-clamped nanobeam (see Figure 5a): normalised transversal displacement against the dimensionless abscissa, x/l, for (**a**) λ=0.1 and (**b**) λ=0.5. Five different values of α were analysed.

Such a normalised displacement is plotted in Figure 6 against the dimensionless abscissa x/l for the five values of the mixture parameter (that is, *α* = 0.00, 0.25, 0.5, 0.75 and 1.00) and two values of the dimensionless characteristic length, that is, λ=0.1 (Figure 6a) and λ=0.5 (Figure 6b).

Analogously, Figure 5b shows a nanobeam of a length l with simply-supported extremities and subjected to a concentrated couple M in the midsection. In such a case, the eight suitable boundary conditions are:

(i) the four kinematic boundary conditions (KBCs) of Equation (20a–d);
(ii) the two static boundary conditions (SBCs) at the nanobeam extremities of Equation (21a,b);
(iii) and the two static boundary conditions (SBCs) at the internal discontinuity point of Equation (22a,b).

Additionally, in this case, the maximum deflection, as the absolute value, $\left|v_{\max}\right|$ is attained at the nanobeam points $x_{1}=\sqrt{3}\cdot l/6$ and $x_{2}=\left(6-\sqrt{3}\right)\cdot l/6$.

The transversal displacement is then normalised with respect to the absolute value of the maximum deflection according to the classical local Bernoulli–Euler solution, that is, $v_{1}^{*}(x)=v_{1}(x)/\left|\sqrt{3}Fl^{2}/216EI\right|$ and $v_{2}^{*}(x)=v_{2}(x)/\left|\sqrt{3}Fl^{2}/216EI\right|$.

**Figure 7:**
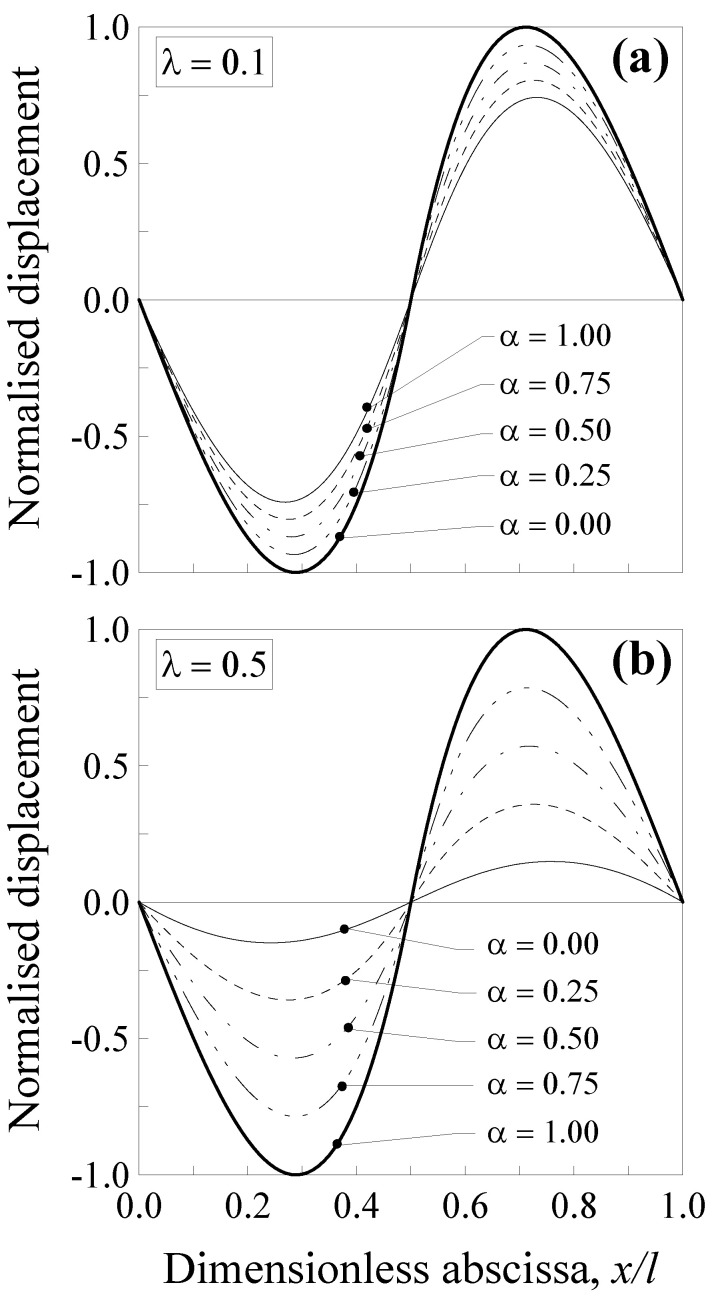
Simply-supported nanobeam (see Figure 5b): normalised transversal displacement against the dimensionless abscissa, x/l, for (**a**) λ=0.1 and (**b**) λ=0.5. Five different values of α were analysed.

Such a normalised displacement is plotted in Figure 7 against the dimensionless abscissa x/l for the five values of the mixture parameter and two values of the dimensionless characteristic length, that is, λ=0.1 (Figure 7a) and λ=0.5 (Figure 7b).

It is interesting to observe that, in the case of the double-clamped nanobeam, the normalised transversal displacement strongly depends on both the mixture parameter, α and the dimensionless characteristic length, λ, showing a decrease of about 52% for λ=0.1 (Figure 6a) and of about 96% for λ=0.5 (Figure 6b) when α=0.0 and x/l=l/3 or x/l=2l/3. In contrast, such a decrease was lower in the case of the simply-supported nanobeam, since it is equal to about 26% for λ=0.1 (Figure 7a) and of about 86% for λ=0.5 (Figure 7b) when α=0.0 and $x/l=\sqrt{3}/6$ or $x/l=\left(6-\sqrt{3}\right)/6$.

Consequently, the nanobeams generally show a stiffer behaviour with respect to large-scale beams.

### 3.3. Nanobeams with a Non-Uniform Distributed Load

**Figure 8:**
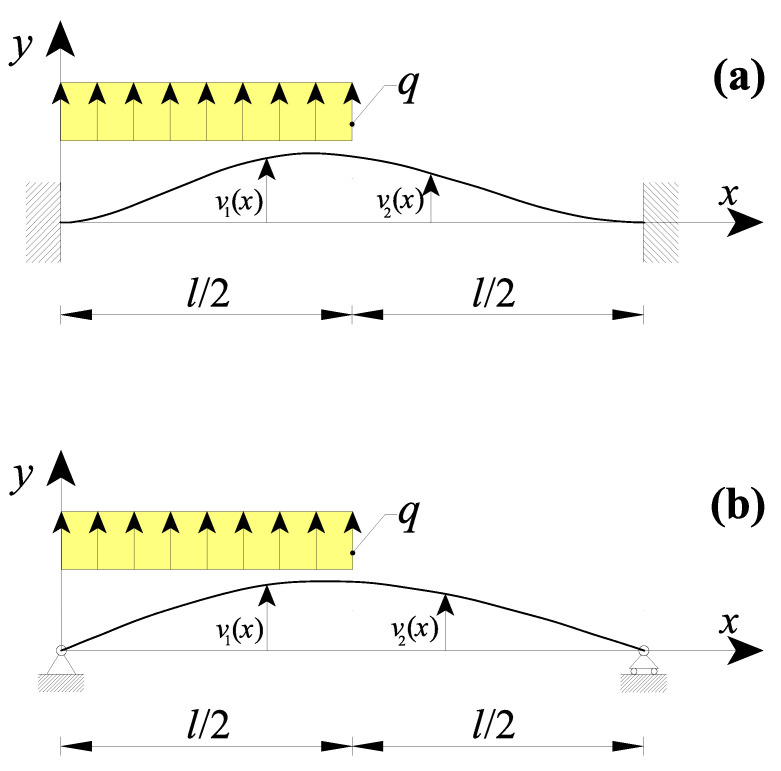
Nanobeam containing a loading discontinuity in the midsection due to a non-uniform distributed load q with the beam ends: (**a**) double-clamped and (**b**) simply-supported.

Finally, the last two case studies considered a nanobeam with a length l subjected to a non-uniform distributed load q (q=const.), as shown in Figure 8.

In particular, in the first case, a double-clamped nanobeam was considered and the internal loading discontinuity was in the midsection (i.e., x=l/2, Figure 8a). According to the above dissertation, the nanobeam transversal displacement was obtained by considering in addition to both Equation (16a,b) (CBCs) and Equation (17a,b) (CCCs), the following conditions:

(i) the six kinematic boundary conditions (KBCs) of Equation (18a–f);
(ii) and two static boundary conditions (SBCs):
  (23a) $$v_{1}^{\left(3\right)}\left(l/2\right)-L_{c}^{2}v_{1}^{\left(5\right)}\left(l/2\right)=v_{2}^{\left(3\right)}\left(l/2\right)-L_{c}^{2}v_{2}^{\left(5\right)}\left(l/2\right)$$
  (23b) $$v_{1}^{\left(2\right)}\left(l/2\right)-L_{c}^{2}v_{1}^{\left(4\right)}\left(l/2\right)+\alpha L_{c}^{2}\frac{q}{IE}=v_{2}^{\left(2\right)}\left(l/2\right)-L_{c}^{2}v_{2}^{\left(4\right)}\left(l/2\right)$$

As can be noted, particular attention is paid to the definition of the second SBC, since, by imposing the continuity of the bending moment at the point x=l/2, the second-order derivative of the bending moment is also added in the equation, with it being different from zero. More precisely, the bending moment is defined, according to Equation (15a,b), as:

(24a) $$M_{1}^{}\left(x\right)=IE\left[v_{1}^{\left(2\right)}\left(x\right)-L_{c}^{2}v_{1}^{\left(4\right)}\left(x\right)+\alpha L_{c}^{2}\frac{M_{1}^{\left(2\right)}\left(x\right)}{IE}\right]$$

(24b) $$M_{2}^{}\left(x\right)=IE\left[v_{2}^{\left(2\right)}\left(x\right)-L_{c}^{2}v_{2}^{\left(4\right)}\left(x\right)+\alpha L_{c}^{2}\frac{M_{2}^{\left(2\right)}\left(x\right)}{IE}\right]$$

Due to the particular loading configuration, $M_{1}^{\left(2\right)}\left(x\right)=q$, whereas $M_{2}^{\left(2\right)}\left(x\right)=0$. Consequently, by equating the two bending moments of Equation (24a,b) for x=l/2, the SBC of Equation (23b) can be obtained.

The maximum deflection $v_{\max}$ was attained at the nanobeam point x≃0.443⋅l.

The transversal displacement was then normalised with respect to the maximum deflection according to the classical local Bernoulli–Euler solution, that is, $v_{1}^{*}(x)=v_{1}(x)/\left(1.34\cdot 10^{-3}ql^{4}/EI\right)$ and $v_{2}^{*}(x)=v_{2}(x)/\left(1.34\cdot 10^{-3}ql^{4}/EI\right)$.

**Figure 9:**
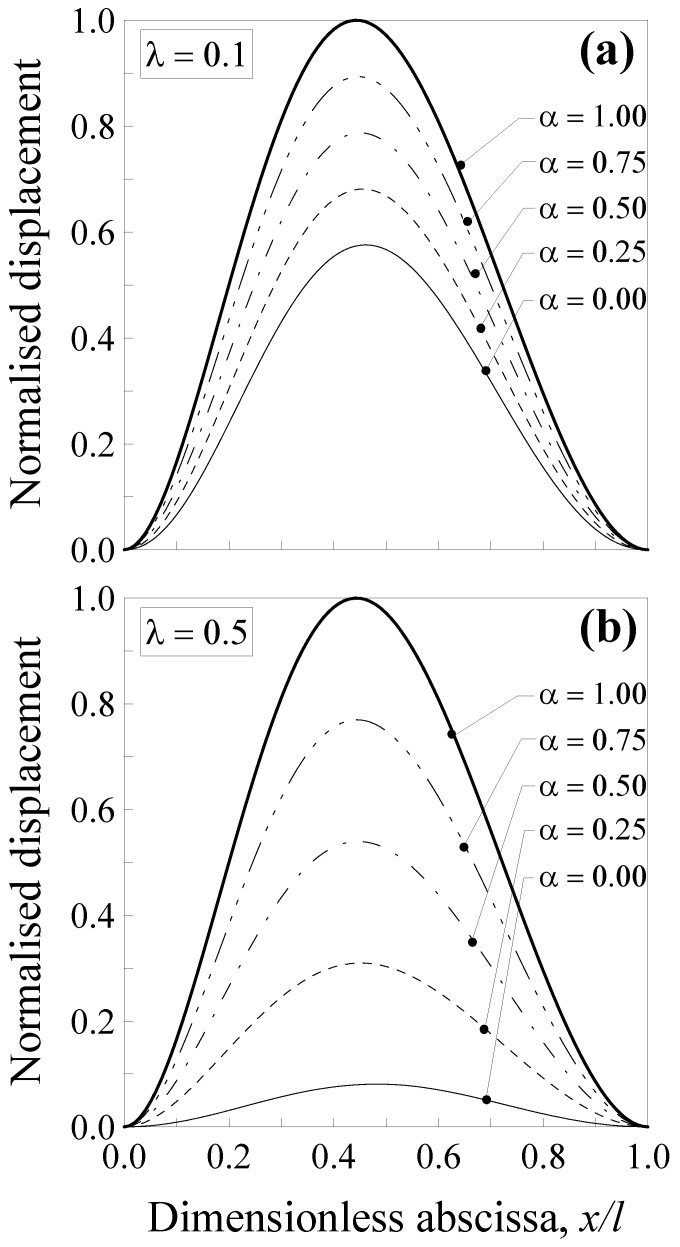
Double-clamped nanobeam (see Figure 8a): normalised transversal displacement against the dimensionless abscissa, x/l, for (**a**) λ=0.1 and (**b**) λ=0.5. Five different values of α were analysed.

Such a normalised displacement is plotted in Figure 9 against the dimensionless abscissa x/l for the five values of the mixture parameter (that is, α=0.00, 0.25, 0.50, 0.75 and 1.00) and two values of the dimensionless characteristic length, that is, λ=0.1 (Figure 9a) and λ=0.5 (Figure 9b).

Analogously, Figure 8b shows a nanobeam of a length l with simply-supported extremities and subjected to a non-uniform distributed load q (q=const.) with internal discontinuity at the point x=l/2. In these cases, the eight suitable boundary conditions are:

(i) the four kinematic boundary conditions (KBCs) of Equation (20a–d);
(ii) two static boundary conditions (SBCs) at the nanobeam extremities:
  (25a) $$v_{1}^{\left(2\right)}\left(0\right)-L_{c}^{2}v_{1}^{\left(4\right)}\left(0\right)+\alpha L_{c}^{2}\frac{q}{IE}=0$$
  (25b) $$v_{2}^{\left(2\right)}\left(l\right)-L_{c}^{2}v_{2}^{\left(4\right)}\left(l\right)=0$$
(iii) and the two static boundary conditions (SBCs) at the internal discontinuity point of Equation (23a,b).

Furthermore, in this case, particular attention was paid to the definition of Equation (25a), since by imposing $M_{1}^{}\left(0\right)=0$, the second-order derivative of the bending moment can also be introduced, with it being different from zero ($M_{1}^{\left(2\right)}\left(x\right)=q$).

The maximum deflection $v_{\max}$ was attained at the nanobeam point x≃0.460⋅l.

The transversal displacement was then normalised with respect to the maximum deflection according to the classical local Bernoulli-Euler solution, that is, $v_{1}^{*}(x)=v_{1}(x)/\left(6.56\cdot 10^{-3}ql^{4}/EI\right)$ and $v_{2}^{*}(x)=v_{2}(x)/\left(6.56\cdot 10^{-3}ql^{4}/EI\right)$.

**Figure 10:**
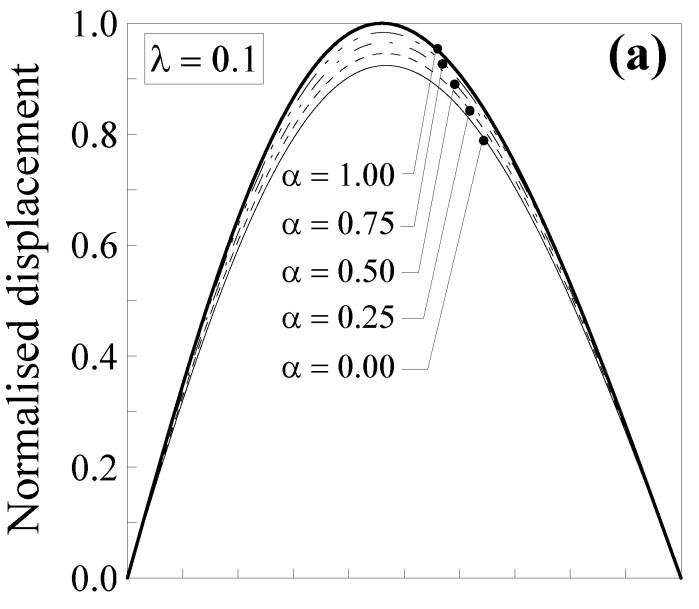

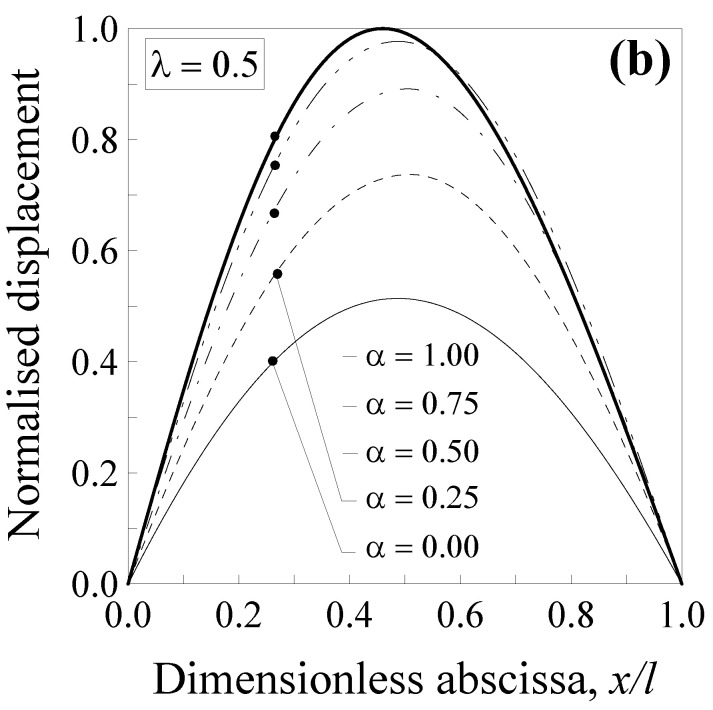
Simply-supported nanobeam (see Figure 8b): normalised transversal displacement against the dimensionless abscissa x/l for (**a**) λ=0.1 and (**b**) λ=0.5. Five different values of α were analysed.

Such a normalised displacement is plotted in Figure 10 against the dimensionless abscissa x/l for the five values of the mixture parameter and two values of the dimensionless characteristic length, that is, λ=0.1 (Figure 10a) and λ=0.5 (Figure 10b).

It is interesting to observe that, in the case of the double-clamped nanobeam, the normalised transversal displacement strongly depends on both the mixture parameter, α and the dimensionless characteristic length, λ, showing a decrease of about 42% for λ=0.1 (Figure 9a) and of about 92% for λ=0.5 (Figure 9b) when α=0.0 and x/l≃0.443. In contrast, such a decrease was lower in the case of the simply-supported nanobeam, since it was equal to about 8% for λ=0.1 (Figure 10a) and of about 49% for λ=0.5 (Figure 10b) when α=0.0 and x/l≃0.460. 

Consequently, the nanobeams generally show a stiffer behaviour with respect to large-scale beams.

## 4. Application: Cracked Nanobeam

**Figure 11:**
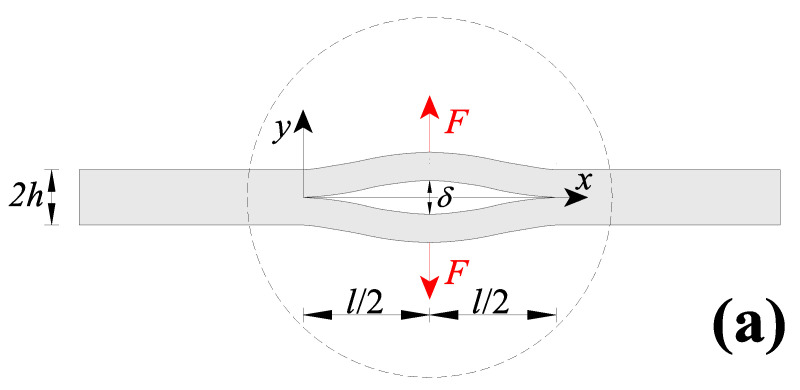

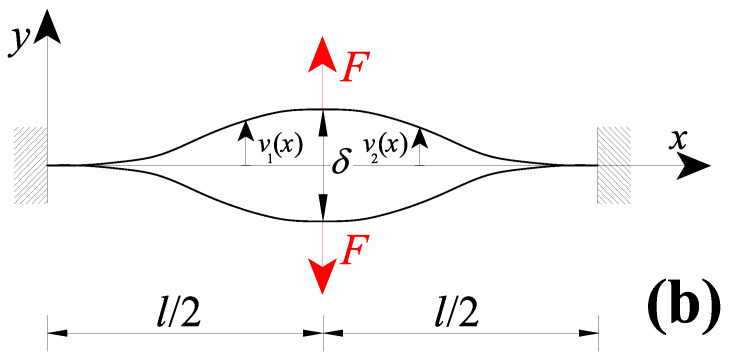
Centrally-cracked nanobeam: (**a**) geometry and (**b**) where the crack is schematised as a double clamped-clamped nanobeam.

In the present section, a centrally-cracked nanobeam (CCN) was considered. The CCN, with a rectangular cross-section with thickness, 2h, and width, b, contains a central crack with length, l, and is subjected to a pair of concentrated forces at the half crack length (Figure 11a).

The presence of the crack may be conveniently modelled by assuming it as a pair of double-clamped nanobeams having a length equal to the cracked one, as shown in Figure 11b [51]. The deformed crack faces of the pair of double-clamped nanobeams under the action of the external loads may be analysed by computing the deflection of the two identical clamped-clamped nanobeams. By taking advantage of the symmetry, a single double-clamped nanobeam was considered (i.e., the upper one in Figure 11b), where the solution of such a problem in terms of transversal displacement was previously presented in Section 3.1.

**Figure 12:**
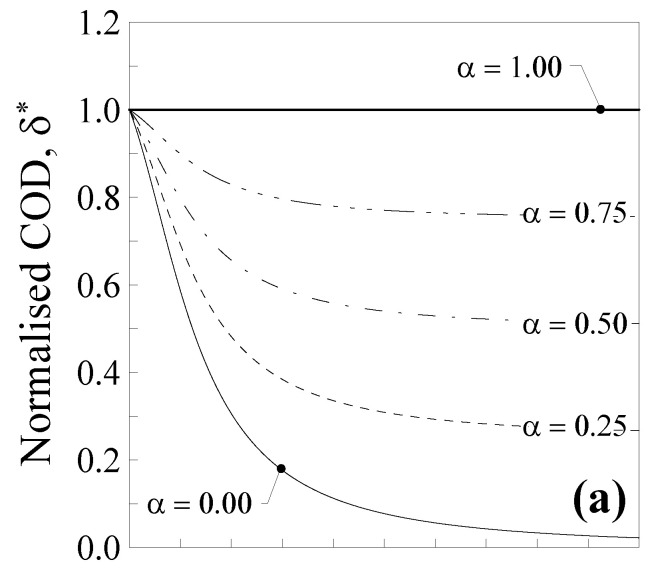

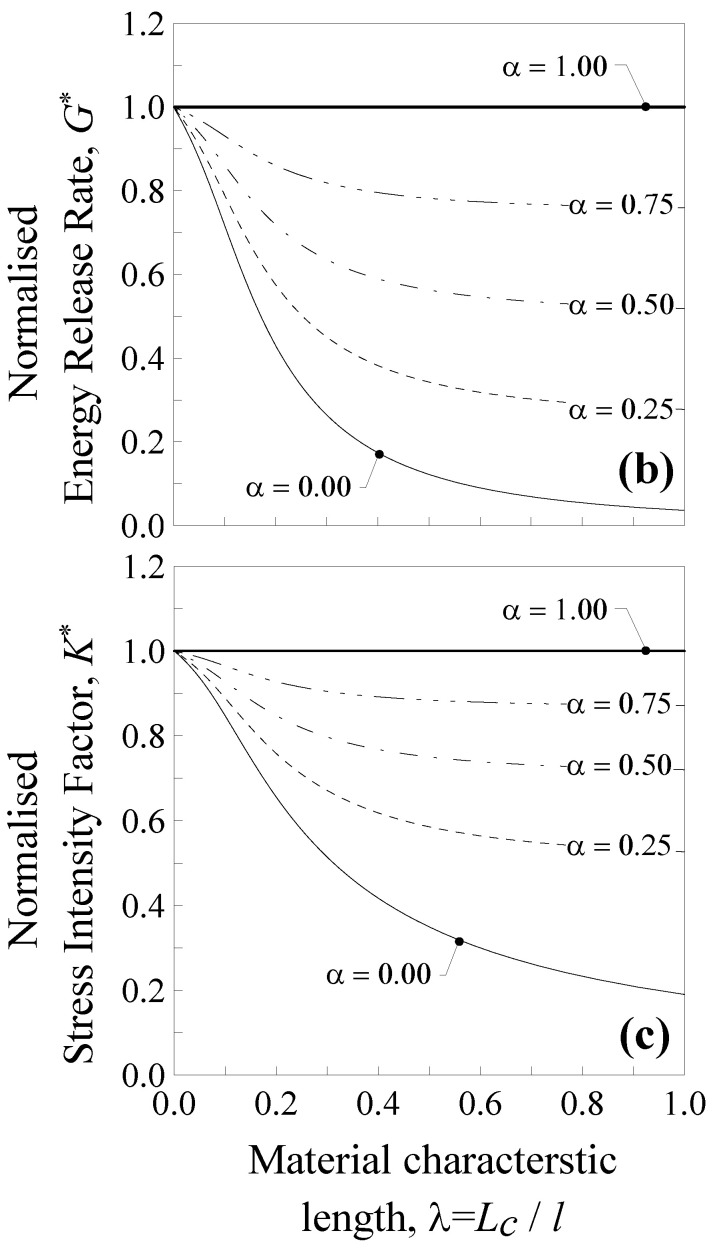
Centrally-cracked nanobeam: (**a**) normalised COD, $\delta^{*}$, (**b**) normalised energy release rate, $G^{*}$, and (**c**) normalised stress intensity factor, $K^{*}$, against the dimensionless characteristic length, λ. Five different values of α were analysed.

The maximum crack opening displacement (COD), δ, may be computed as two-times the deflection at the half length of the double-clamped nanobeam, that is, $\delta =2\cdot v_{1}(l/2)=2\cdot v_{2}(l/2)$. The COD is conveniently normalised with respect to the well-known classical local Bernoulli–Euler solution, that is, $\delta^{*}=\delta /\left(2\cdot \frac{Fl^{3}}{192I_{E}}\right)$, and plotted in Figure 12a against the dimensionless characteristic length λ for five values of the mixture parameter (that is, *α* = 0.00, 0.25, 0.50, 0.75 and 1.00).

As can be observed, the normalised COD, $\delta^{*}$, decreases by increasing the value of λ for all the considered values of the mixture parameter, with the exception of α=1.0 (thick line), for which $\delta^{*}=1.0$ (local Bernoulli–Euler problem). The nanobeam also behaves like a large scale beam when λ=0.0, whereas the effect of nonlocality is maximum when λ=1.0 and α=0.0 (thin continuous line), with a maximum decrease in the COD equal to about 95%, corresponding to the case of the full nonlocal model. Consequently, it is possible to observe that in a centrally-cracked nanobeam, the crack is less open than in a large-scale one.

Finally, by increasing α from 0.0 to 1.0, $\delta^{*}$ progressively increases up to the limit value equal to 1.0 (pure local problem).

### Energy Release Rate and Stress Intensity Factor

The energy release rate per unit width (or crack extension force), ERR, is determined by deriving the total strain energy stored in the deformed cracked nanobeam with respect to the crack length l:

(26) $$G=\frac{1}{b}\frac{dW}{dl}$$

with the work done by the two external forces, F, expressed as:

(27) $$W=2\left[\frac{1}{2}Fv_{1}(l)\right]=2\left[\frac{1}{2}Fv_{2}(l)\right]$$

The above energy release rate of Equation (26) is then normalised with respect to the classical local solution, $G_{C}=\left(F^{2}l^{2}\right)/\left(64bEI\right)$, so that:

(28) $$G^{*}=\frac{G}{\left(F^{2}l^{2}\right)/\left(64bEI\right)}$$

Such a normalised ERR is then plotted in Figure 12b against the dimensionless characteristic length, λ , for different values of α . It is interesting to note that the normalised ERR, $G^{*}$, always decreases by increasing λ for all the considered values of the mixture parameter, with the exception of α=1.0 (thick line), for which $G^{*}=1.0$ (i.e., local Bernoulli–Euler problem). This decrease was quicker for small values of α, reaching its maximum equal to about 94% when α  was equal to 0.0 (thin continuous line) and λ tending to unity 1.0. From Figure 12b, it is possible to conclude that, in a centrally-cracked nanobeam (λ≠0.0 and α≠1.0), the energy delivered for an additional crack of size dl is lower than that corresponding to a crack in a large-scale centrally-cracked beam, where the crack growth also depends on the material characteristic length, $L_{c}$.

Finally, the stress intensity factor (SIF) for CCN is computed by exploiting the its relationship with the energy release rate per unit width by assuming, for the sake of simplicity, a plane stress state:

(29) $$K=\sqrt{EG}$$

where *E* is the elastic modulus of the material.

Even in this case, it is more convenient to introduce a normalised quantity by dividing the above SIF by the well-known classical local Bernoulli–Euler solution, $K_{C}=\left(Fl/8\right)\sqrt{{\left(bI\right)}^{-1}}$, as:

(30) $$K^{*}=\frac{K}{\left(Fl/8\right)\sqrt{{\left(bI\right)}^{-1}}}$$

In Figure 12c, the normalised SIF is plotted against λ for different values of α . Similar comments may be made for $K^{*}$ and the maximum decrement (of 80%) was observed when α=0.0 and λ tends to 1.0 (thin continuous line).

## 5. Conclusions

In the present paper, the two-phase local/nonlocal stress-driven integral model was extended in order to consider the internal discontinuities caused, for instance, by concentrated forces and/or couples, non-uniform distributed loads, discontinuity in the beam geometry, internal supports/constrains, and so on. To this aim, the original two-phase local/nonlocal SDM formulation, which avoids internal discontinuities, was revised.

The presented formulation was validated by considering six case studies, that is, two nanobeam constrain configurations (i.e., double-clamped and simply-supported nanobeams) and for each of them, three loading configurations (i.e., a concentrated force and a couple applied at the nanobeam midsection, and a non-uniform distributed load). For the above cases, the displacement field was determined and the effect of nonlocality by means of both the dimensionless characteristic length and mixture parameter was discussed by observing that:

(i) high degree of nonlocality, that is, greater values of the dimensionless characteristic length and small values of the mixture parameter cause a stiffer behaviour of the nanobeam with respect to the large-scale counterpart, especially for the double-clamped nanobeams;
(ii) by increasing the value of the mixture parameter up to the unit, the nanobeam behaves as a large-scale beam according to the classical local Bernoulli–Euler model; and
(iii) the combined use of both mixture parameter and dimensionless characteristic length allows us to improve the applicability of the SDM. This is possible, since, while the dimensionless characteristic length depends on the material microstructures through the characteristic length, *L_c_*, and this parameter is a constant for a nanobeam with a fixed length and made of a given material, so the mixture parameter may be calibrated in order to properly describe the behaviour of real nanostructures.

Finally, a centrally-cracked nanobeam, subjected to concentrated forces at the crack half-length, was studied. The crack opening displacement was computed by varying both dimensionless characteristic length and mixture parameter values, whereas the energy release rate and the stress intensity factor were determined by employing the compliance method and the linear elastic fracture mechanics concepts. From the obtained results, it was observed that the analysed fracture properties vary significantly with the dimensionless characteristic length of the material, manifesting the so-called small-scale effect. As a matter of fact, the crack growth depends not only on the crack length (like in a large-scale cracked beam), but also on the characteristic length of the material. Moreover, the energy release rate strongly decreases by increasing the nonlocality, showing the potential superior fracture performance of nanobeams with respect to large-scale beams.
